# Consumption of ultra-processed foods and associated factors in children from Barbacena (MG), Brazil

**DOI:** 10.1590/1984-0462/2024/42/2022127

**Published:** 2023-05-15

**Authors:** Marlene de Melo Fonseca, Rafaela Vitoria Guilherme Coimbra, Julia Silva e Oliveira, Anne Danieli Nascimento Soares, Júnia Maria Geraldo Gomes

**Affiliations:** a Instituto Federal de Educação, Ciência e Tecnologia do Sudeste de Minas Gerais, Barbacena, MG, Brazil.; a Universidade Federal de Viçosa, Viçosa, MG, Brazil.

**Keywords:** Child, Diet surveys, Feeding behavior, Food intake, Industrialized foods, Criança, Pesquisa alimentar, Comportamento alimentar, Ingestão alimentar, Alimentos industrializados

## Abstract

**Objective:**

To evaluate the prevalence of ultra-processed food consumption and associated factors among children enrolled in the public school system of the city of Barbacena, Minas Gerais, Brazil.

**Methods:**

This is a cross-sectional study conducted with schoolchildren aged 7–9 years, of both sexes, enrolled in state public schools. Food intake was assessed using the Previous Day Food Questionnaire and the level of physical activity by the Previous Day Physical Activity Questionnaire. The listed foods were classified according to the extent and purpose of industrial processing, using the NOVA classification. Pearson’s χ^2^ test, Fisher’s exact test, χ^2^ with Yates correction, and Poisson regression were used in the statistical analysis, estimating the crude and adjusted prevalence ratio, with 95% confidence intervals.

**Results:**

The prevalence of daily ultra-processed food consumption was 69.6%. After adjusted analyses, the consumption of ultra-processed food was associated with the omission of breakfast, mid-afternoon snack, supper, low physical activity, and consumption of risk foods. On the other hand, consumption of *in natura* or minimally processed foods was associated with older age, the consumption of lunch, mid-afternoon snack, dinner, and protective foods.

**Conclusions:**

There is a high prevalence of ultra-processed foods consumption, associated with unhealthy dietary habits among schoolchildren. This highlights the need for nutritional counseling and educational actions, favoring healthy eating in childhood.

## RESUMO

**Objetivo:**

Avaliar a prevalência de consumo de alimentos ultraprocessados e fatores associados em crianças matriculadas na rede pública do município de Barbacena, Minas Gerais, Brasil.

**Métodos:**

Trata-se de um estudo transversal realizado com escolares de 7 a 9 anos de ambos os sexos, matriculados na rede pública estadual de ensino. O consumo alimentar foi avaliado utilizando o Questionário Alimentar do Dia Anterior e o nível de atividade física pelo Questionário de Atividade Física do Dia Anterior. Os alimentos listados foram classificados segundo a extensão e propósito do processamento industrial, conforme proposto pela classificação NOVA. Na análise estatística foram utilizados os testes χ^2^ de Pearson, Exato de Fisher, χ^2^ com correção de Yates e regressão de Poisson, estimando-se a razão de prevalência bruta e ajustada, com intervalos de confiança de 95%.

**Resultados:**

A prevalência de consumo de alimentos ultraprocessados foi de 69,6%. Após análises ajustadas, o consumo de alimentos ultraprocessados foi associado à omissão do café da manhã, lanche da tarde e ceia, baixo nível de atividade física e ao consumo de alimentos de risco. Por outro lado, o consumo de alimentos *in natura* ou minimamente processados foi associado à maior idade, consumo de almoço, lanche da tarde, jantar e alimentos protetivos.

**Conclusões:**

Verificamos uma alta prevalência no consumo de alimentos ultraprocessados, associada a hábitos alimentares não saudáveis, em crianças escolares. Isso destaca a necessidade de ações de educação alimentar e nutricional, favorecendo o consumo alimentar saudável na infância.

## INTRODUCTION

Obesity and overweight are considered a global epidemic, which affects not only adults but also children and adolescents.^1,2^ The World Obesity Federation estimates that 71 million children aged 5–9 years are obese nowadays. In Brazil, it is predicted that 7.7 million children will be obese by 2030.^3^

The increased prevalence of obesity in childhood is associated with changes in behavioral factors, like poor physical activity, inadequate sleep, watching too much TV, and eating habit, such as skipping breakfast and drinking sugar-sweetened beverages.^4^ In addition, consumption of ultra-processed foods was associated with adiposity, worsening of the lipid profile and metabolic syndrome in childhood and adolescence.^5,6,7,8^ According to the Family Budget Survey, 19.7% of the calories consumed by the Brazilian population over ten years old consisted of ultra-processed food. Data from the National School Health Survey show that 97.3% of schoolchildren aged between 13–19 years consumed at least one ultra-processed food daily.^9,10^

Ultra-processed foods are products made up by industry, combining ingredients extracted plus synthesized substances.^11^ Evidence from the literature emphasizes that the high consumption of ultra-processed foods by children may lead to a lower intake of several nutrients as n-3 polyunsaturated fatty acids, fiber, vitamins (A, B12, C, and E) and minerals (Ca, K, and Zn), which may lead to impaired growth and affect adulthood and the elderly.^12^

Although studies have evaluated the consumption of ultra-processed food in schoolchildren,^13,14^ only a few studies assessed the consumption of ultra-processed food in Brazilian children aged 7–9 years, focusing on meal patterns. In a previous study conducted by our group, we verified a high prevalence of excessive body weight in children aged 7–9 years (more than 30%) in Barbacena (MG).^15^ We also observed that this population is vulnerable to unhealthy dietary patterns, which increases the risk for obesity.^15^ Thus, considering the high risk of excessive body weight in children living in this area and the hyper-palatability and ease of consumption of ultra-processed foods,^16^ the evaluation of these schoolchildren is relevant since the consumption of the ultra-processed might be high.^17^ In view of this, we aimed to assess the prevalence of consumption of ultra-processed food and factors associated, focusing on meal pattern, among children aged 7–9 years from state public schools in Barbacena (MG).

## METHOD

The study protocol was approved by the Ethics Committee for Research with Human Beings of the Federal Institute of the Southeast of Minas Gerais (No. 3.588.082), according to the recommendations of the Declaration of Helsinki. The participating children signed the free and informed assent term, and their guardians signed the free and informed consent form.

This is a cross-sectional study carried out with schoolchildren aged 7–9 years, of both sexes, enrolled in the state public school system of the city of Barbacena (MG), carried out between July 2019 and February 2020. This age range was selected due to the scarcity of studies covering this period.^15^

According to the State Superintendence of Education of Barbacena, based on the 2018 school census in the urban area, there were 2,823 students from 1st to 5th grade of elementary school in the state public network. The sample size was calculated using a 95% confidence interval (95%CI), sampling error of 5%, and prevalence of overweight in schoolchildren of 37%, corresponding to the minimum sample size of 318 schoolchildren. At that time, there were 76 schools in the city, and of them, 12 belonged to the state public system. First, a cluster sampling was carried out, and four schools were chosen by random drawing in each region of the city (two central and two peripheral schools). In these four schools, there were 1,148 students enrolled in the 1st to 5th grades. Next, the selection of students per school and these grades was carried out by simple random drawing until the required number of students was completed.

We assessed all students who were 7–9 years old in these schools (n=330). However, data from children with special needs, such as neurological disorder (n=3), or physical disability (n=1), were not included in the statistical analyses. All the evaluated schoolchildren studied in the afternoon shift.

Four trained researchers applied the questionnaires. To assess food intake, we used the Previous Day Food Questionnaire (QUADA) version 3, an illustrated and structured instrument validated for schoolchildren.^18^ The questionnaire was structured into six meals (breakfast, mid-morning snack, lunch, mid-afternoon snack, dinner, and supper) with 21 foods or food groups each. The researchers applied the questionnaire according to the method adapted from the application manual of the QUADA and Previous Day Physical Activity Questionnaire (QUAFDA) of the Federal University of Santa Catarina.^19^ Briefly, six booklets identified by the type of meal were presented, one time. If necessary, the names of each food illustrated were passed on. First, the children were asked to remember what they ate at each specific time. Thus, the researchers circled the foods they indicated.

Food groups of QUADA are classified into protective foods (fruits, natural juices, vegetables and greens, and vegetable soup) and risk foods (fast-food, chocolate milk, sweets and desserts, chips, industrialized snacks, soft drinks, and artificial fruit juice).^20^ For protective foods, the consumption was considered adequate when it was more frequently, or equal to six times a day, and inadequate when it was five times a day or less. The risk foods were classified as adequate when consumed twice a day or less, or inadequate when consumed more than three times a day.^21,22^

The listed foods were classified according to the extent and purpose of industrial processing, as proposed by the NOVA classification. Thus, the foods were divided into three groups:

1. *In natura* or minimally processed,
2. Processed, and
3. Ultra-processed foods.^11^

Levels of physical activity were assessed using the QUAFDA,^18,23^ according to the same instruction manual.^19^ The questionnaire consists of an illustrated booklet with various activities (dancing, walking, running, playing with animals, doing household chores, riding a bicycle, etc.). First, the children were requested to recall their physical activities from the previous day. Concepts of intensity (slow, fast, and very fast) were explained to the children, using physiological indicators such as breathing, heart rate, and perspiration. Each activity illustrated was named, taking into account the intensities. Then, children were requested to indicate each physical activity and intensity performed the previous day. For classification, attributes were used according to the intensity of physical activity, and the students were classified as little active, moderately active, and very active.^23^

Data were tabulated in Microsoft Office Excel^®^ and analyzed using Statistical Package for Social Sciences (SPSS software^®^), version 20.0.

Categorical variables (age group, sex, geographic location of the school, physical activity level, meals consumed, consumption of *in natura* or minimally processed foods, and ultra-processed foods) were evaluated by Pearson’s χ^2^ test, Fisher’s exact test, and χ^2^ with Yates correction. Poisson regression with robust variance was used to obtain the crude and adjusted prevalence ratio (PR) to verify the association between the consumption of *in natura* or minimally processed foods and ultra-processed foods with the independent variables. The dependent variables were presence or absence, and independent variables were age, geographic location of the school, physical activity level, consumption or absence of meals, and consumption of protective and risk foods. We chose this type of modeling because it is preferable in cross-sectional studies, since odds ratio (OR) from logistic regression may overestimate the prevalence ratio.

All variables with p<0.20 were included in the adjusted regression. A significance level of 95% (p≤0.05) was considered.

## RESULTS

A total of 326 children aged 7–9 years from four state public schools in Barbacena (MG), participated in this study. Anthropometric and body composition results have been previously published.^15^ Most of the sample was female (51.8%) and studied in central schools (61%). Almost all (98.8%) of the students were little active. Regarding the number of daily meals, most of the students had the main ones: breakfast (86.2%), lunch (94.2%), mid-afternoon snack (85%), and dinner (86.8%). However, the habit of having mid-morning snack (79.8%) and supper (56.4%) was less frequent (Table 1).

**Table 1.** Characteristics of the sample of schoolchildren participating in the study (n=326). Barbacena (MG), Brazil, 2021.

Variable	Total	
	n	%
Sex		
Female	169	51.8
Age group (years)		
7 to <8	112	34.4
8 to <9	102	31.3
9 to <0	112	34.4
Geographic location of the school		
Central	199	61.0
Meal consumption (yes)		
Breakfast	281	86.2
Mid-morning snack	66	20.2
Lunch	307	94.2
Mid-afternoon snack	277	85.0
Dinner	283	86.8
Supper	142	43.6
Daily consumption of at least one ultra-processed food		
Yes	227	69.6
Physical activity practice		
Little active	322	98.8
Moderate active	4	1.2
Very active	0	0.0
Protective foods		
Inadequate	312	95.7
Risk foods		
Adequate	200	61.3

Data expressed as absolute and relative frequency.

Although the prevalence of daily consumption of *in natura* or minimally processed food was high (66.9%), almost all schoolchildren (95.7%) reported inadequate consumption of protective food (fruits, vegetables, and legumes), which means equal or less than 5 times a day (Table 1). The prevalence of consumption of *in natura* or minimally processed food is higher among those who had adequate consumption of protective food (100 *vs.* 65.4%; p=0.006), and among those who consume risk foods less frequently, e.g., adequate consumption (71.5 *vs.* 59.5%; p=0.030) (Table 2).

**Table 2.** Prevalence of consumption of unprocessed, processed, and ultra-processed foods according to gender, geographic location, meals eaten, practice of physical activity, and consumption of protective and risk foods by schoolchildren (n=326). Barbacena (MG), Brazil. 2021.

Variables	*In natura* or minimally processed food consumption			Ultra-processed food consumption		
	Present		p-value	Present		p-value
	n	%		n	%	
Sex						
Female	117	69.2	0.41	116	68.6	0.719
Male	101	64.3		111	70.7	
Geographic location of the school						
Central	130	65.3	0.472	136	68.3	0.540
Peripheral	88	69.3		91	71.7	
Consumed meals according to QUADA						
Breakfast						
Yes	195	69	**0.018**	204	72.6	**0.005**
No	23	51		23	51.1	
Mid-morning snack						
Yes	48	72.7	0.306	52	78.8	0.074
No	170	47.2		175	67.3	
Lunch						
Yes	214	69.7	**0.000**	214	69.7	1.000
No	4	21.1		13	68.4	
Mid-afternoon snack						
Yes	192	69.3	**0.032**	204	73.7	**0.000**
No	26	53.1		23	46.9	
Dinner						
Yes	202	71.4	**0.000**	199	70.3	0.594
No	16	37.2		28	65.1	
Supper						
Yes	104	76.5	**0.033**	110	77.5	**0.008**
No	114	62.0		117	63.6	
Physical activity level						
Little active	216	67.1	0.602	223	69.3	0.318
Moderate and very active	2	50.0		41	100.0	
Protective foods						
Adequate	14	100.0	**0.006**	8	57.1	0.373
Inadequate	204	65.4		219	70.2	
Risk foods						
Adequate	143	71.5	**0.030**	101	50.5	**0.000**
Inadequate	75	59.5		126	100.0	

Data is expressed as absolute and relative frequency. Pearson’s χ^2^. Fisher’s Exact and Yates’ correction χ^2^ tests were performed. Bold type letters indicated significant differences (p<0.050). QUADA: Previous Day Food Questionnaire.

Children who consumed breakfast (69.4 *vs.* 51.1%; p=0.018), lunch (69.7 *vs.* 21.1%; p=0.000), mid-afternoon snack (69.3 vs. 53.1%; p=0.032), dinner (71.4 *vs.* 37.2%; p=0.000) and supper (73.2 *vs.* 62.0%, p=0.033) reported higher consumption of *in natura* or minimally processed foods compared with those who did not consume such meals (Table 2).

Almost 70% of children related the daily consumption of at least one ultra-processed food (Table 1). The inadequate consumption of risk foods, such as cookies, soft drinks, snacks, French fries, and pizza, more than three times a day, was reported by 38.6% of the schoolchildren (Table 1). The consumption of ultra-processed foods was higher among the schoolchildren who presented inadequate consumption of risk foods (100 vs. 50.5%; p=0.000) (Table 2).

In addition, the schoolchildren who ate breakfast (72.6 *vs.* 51.1%; p=0.005), mid-afternoon snack (73.6 *vs.* 46.9%; p=0.000) and supper (77.5 *vs.* 63.6%; p=0.008) had higher prevalence of consumption of ultra-processed foods (Table 2).

We observed that age (PR 1.05; 95%CI 1.01–1.09), consumption of lunch (PR 0.78; 95%CI 0.70–0.88), mid-afternoon snack (PR 0.86; 95%CI 0.78–0.95), dinner (PR 0.77; 95%CI 0.71–0.87) and adequate consumption of protective foods (PR 0.88; 95%CI 0.82–0.95) were associated with the consumption of *in natura* or minimally processed foods, even after adjustments. Skipping supper was associated with higher consumption of *in natura* foods after adjustments (PR 1.10; 95%CI 1.02–1.18) (Table 3).

**Table 3.** Crude and adjusted analyses of the prevalence ratio of daily consumption of unprocessed, processed, and ultra-processed foods according to age, meals eaten, physical activity, and consumption of protective and risk foods.

Variables	Consumption of *in natura* or minimally processed food		Consumption of ultra-processed foods	
	PR (95%CI)	aPR (95%CI)	PR (95%CI)	aPR (95%CI)
Age (years)				
7 to 8.4	1	1	–	–
8.5 to 9	1.09 (1.03–1.15)	1.05 (1.01–1.09)	–	–
Breakfast				
Yes	1	–	1	1
No	0.90 (0.80–0.99)	–	1.11 (1.03–1.20)	1.14 (1.06–1.22)
Lunch				
Yes	1	1	–	–
No	0.77 (0.68–0.87)	0.78 (0.70–0.88)	–	–
Mid-afternoon snack				
Yes	1	1	1	1
No	0.87 (0.79–0.95)	0.86 (0.78–0.95)	1.17 (1.07–1.23)	1.13 (1.04–1.23)
Dinner				
Yes	1	1	–	–
No	0.78 (0.71–0.86)	0.77 (0.71–0.87)	–	–
Supper				
Yes	1	1	1	1
No	1.11 (1.03–1.19)	1.10 (1.02–1.18)	1.11 (1.04–1.18)	1.11 (1.04–1.18)
Consumption of protective foods				
Adequate	1	1	–	–
Inadequate	0.86 (0.78–0.95)	0.88 (0.82–0.95)	–	–
Consumption of risk foods				
Adequate	1	–	1	1
Inadequate	1.11 (1.03–1.19)	–	1.47 (1.39–1.46)	1.45 (1.40–1.55)
Physical activity				
Little active	–	–	1	1
Moderate or very active	–	–	0.84 (0.72–0.98)	0.84 (0.72–0.97)

PR: crude prevalence ratio; aPR: adjusted prevalence ratio; 95%CI: 95% confidence interval. Poisson regression with robust variance was performed to obtain prevalence ratio values.

The omission of breakfast (aPR 1.14; 95%CI 1.06–1.22), mid-afternoon snack (aPR 1.13; 95%CI 1.04–1.23) and supper (aPR 1.11; 95%CI 1.04–1.18), and inadequate consumption of risk foods (aPR 1.45; 95%CI 1.40–1.55) were risk factors for higher consumption of ultra-processed foods, after adjustments (Table 3). On the other hand, being moderate or very active was associated with a lower prevalence of consumption of ultra-processed foods (aPR 0.84; 95%CI 0.72–0.97).

## DISCUSSION

The main results of this study were that consumption of ultra-processed foods was positively associated with omission of breakfast, mid-afternoon snack, supper, consumption of risk foods, and low physical activity in schoolchildren aged 7–9 years. This corroborates previous results observed in adolescents,^24^ in which inappropriate eating context at breakfast and dinner were associated with increases of 2.55% and 4.18%, respectively, in the energy intake derived from ultra-processed foods. Thus, the eating context plays an important role in choosing the type of food consumption based on the processing level.

In this study, we observed a high prevalence (69.6%) of daily consumption of ultra-processed foods among schoolchildren from Barbacena (MG). In younger children (2–5 years old), the National Survey of Children’s Food and Nutrition^25^ observed a very high prevalence of ultra-processed consumption (93%) in Brazil, being even higher in the Southeast region (95%).^25^ However, in another study, the estimated prevalence was 41.0% of daily consumption of foods with low nutritional value among 3,930 schoolchildren from 7 to 10 years old.^26^ Differences such as socio-cultural and economic diversities, geographic locations, the inclusion of children from different age groups, and methods to evaluate the food intake may explain the divergence among studies.

Because ultra-processed foods are high energy-dense, hyper-palatable and poor in micronutrients, the high consumption of these products was associated with an increased risk of chronic diseases in childhood and adulthood.^5–8,11^ However, there is no cut-off for ultra-processed food consumption for children. An interesting study showed that Brazilians of all ages living in households with high consumption of ultra-processed foods (average 564 kcal/day) are 37% more likely to be obese than those with less consumption (average 220 kcal/day).^16^ In our study, we were not able to estimate the calories consumed by this group, but it was estimated that students between 8 and 12 years old in another city of Minas Gerais consumed an average of 501 kcal/day (25% of the total energy).^13^ Considering the similarities in age and geographic location, it is reasonable to assume that the caloric value of this group in our sample is also significant and may be a risk factor for overweight.

On the other hand, consumption of *in natura* or minimally processed foods was associated with older age, consumption of lunch, mid-afternoon snack, dinner and protective foods. The evening meal was a risk factor for lower consumption of *in natura* or minimally processed foods. Although 68.9% of the children assessed consumed *in natura* or minimally processed foods daily, we verified a very high prevalence of inadequacy (more than 95%), e.g., children eat fewer servings per day than recommended, similar to other studies.^10,27^

This highlighted the need for greater attention to the degree of processing of food consumed by schoolchildren. We suggest the adoption of nutritional education actions based mainly on the Food Guide for the Brazilian Population (2014) and the Ten Steps for a Healthy Diet (2014), which:

1. Encourage a diet based on *in natura* or minimally processed foods,
2. Advise against the consumption of ultra-processed foods, and
3. Provide additional advice about a healthy eating context. Prevention measures against chronic diseases should be started from the beginning of life, once they occur from the accumulation of years of a poor diet, especially with exaggerated consumption of ultra-processed foods.

We observed that schoolchildren aged 8.5 to 9 years and 11 months showed a higher prevalence of consumption of *in natura* foods compared to children aged 7 to 8.5 years. Other authors also verified higher proportions of habitual consumption of healthy eating markers among older age students.^10^ As children get older, they understand the importance of healthy eating, which may influence their preferences for foods that add value to their health. We observed a higher prevalence of *in natura* food at lunch and dinner due to the characteristics of the Brazilian diet (e.g., meals with rice, beans, a protein source, and salad).^10^

Traditional mid-afternoon snacks are usually rich in ultra-processed foods, like snack chips and stuffed cookies. However, in our study, eating mid-afternoon snack was associated with higher consumption of *in natura* foods, while omitting this meal was associated with higher consumption of ultra-processed foods. We hypothesized that it is due to the children having their nutritionally balanced lunch and mid-afternoon snack at school, which follow the National School Food Program (PNAE), Law n. 11,947/2009. This program advocates sustainable purchase initiatives, linked to the strengthening of family farming and the Food and Nutrition Security of students, resulting in healthier meals for children.

Children who ate two and three school meals daily consumed 18% and 26% less ultra-processed food, respectively, than those who did not eat any meal.^28^ Although the efficiency of PNAE has increased in recent years due to updates, the program can still present failures such as:

1. Inadequacy of the menus for the needs of each age group,
2. Low use of the resource passed on by the government in family farming purchases,
3. Difficulty in implementing the School Feeding Council, and even in,
4. Hiring nutritionists.

Thus, the monitoring and evaluation of the effectiveness of PNAE should be increased, as well as the children should be encouraged to consume the meals offered.^29^

In the present study, children who ate supper had a higher prevalence of consumption of both *in natura* and ultra-processed foods compared to those who skipped supper. We believe that this controversy is due to the parents’ dietary choices since they may choose more or less healthy meals, depending on their cooking skills and tiredness.^30^

Our strengths are the age group evaluated because they are aware of their school stage and there are few studies in Brazil doing this analysis, in addition, the instrument used in the analysis of food intake and physical activity is specific for schoolchildren.^26^ Conversely, our limitations are the data representing only the consumption made on a single day, not identifying the exact amounts of food ingested, only the type or nutritional quality, and the number of meals in which they were consumed during that day. Another relevant limitation is that the instrument QUADA, used to assess food intake, was not designed to identify degrees of food processing, preceding the publication of the NOVA classification. However, we were able to classify the listed foods following the recommendations of Monteiro et al.^11^ according to the processing level, and into protective and risk groups, which also represent points of congruence with the NOVA classification. Besides, the cross-sectional design does not make it possible to establish cause and effect relationships.

We concluded that the consumption of ultra-processed food presented high prevalence, being consumed more than 3 times a day by more than one-third of the schoolchildren. Skipping breakfast and mid-afternoon snack, and eating supper are risk factors for increased consumption of ultra-processed food, as well as being little active. On the other hand, age, consumption of lunch, mid-afternoon snack, dinner and protective foods were associated with the consumption of *in natura* or minimally processed foods. The evening meal was a risk factor for lower consumption of *in natura* or minimally processed foods. Daily consumption prevalence of *in natura foods* was high but in smaller portions than necessary. These results may contribute to the school environment and government planning of nutritional education actions, reinforcing an application of the PNAE with regard to the planning and implementation of nutritionally balanced menus.

## Data Availability

The database that originated the article is available with the corresponding author.
